# Development of a carer psychoeducational group in an early intervention in psychosis service

**DOI:** 10.1192/j.eurpsy.2025.2171

**Published:** 2025-08-26

**Authors:** A. Gutierrez Vozmediano, M. Carter, J. Lodge

**Affiliations:** 1 South West London and St George’s NHS Mental Health Trust, London, United Kingdom

## Abstract

**Introduction:**

One of the quality statements for the treatment of psychosis in adults refers to the provision of education programmes for carers (NICE QS 80 2015). Our Early Intervention in Psychosis Service (EIS) in South London offered individual support for carers, but there was a need for a structured psychoeducational group for carers.

**Objectives:**

1. The development of a psychoeducational and support programme for carers. 2. A reduction on carers’ experience of burden, as measured by a reduction on the Brief Experience of Caregiving Inventory (BECI). 3. An improvement on the carers’ wellbeing, as measured by an increase on the Warwick-Edinburgh Mental Well-being Scale (WEMWBS).

**Methods:**

The team used the materials created by the University of Lancaster in REACT (Lobban *et al.* BJPsych; 2013 203 366-72), and further amended them to reflect the local services, and expand peer support discussions. Sessions were further co-produced with input from team members, and following feedback from participants. Quantitative feedback was obtained before the group and in the end. Qualitative feedback about the group’s experience was elicited at each session.

**Results:**

The group was attended by 7.1 participants on average, with a drop out of 3 participants after the first session. For those participants that completed the group, it was elicited an improvement on the experience of burden associated to caregiving and on wellbeing; please see Table 1 for details. The improvement on BECI included its four subscales: Stigma/Effects on Family, Positive Personal Experiences, Problems with Services and Difficult Behaviours. Qualitative feedback elicited that the participants felt listened to, their knowledge about psychosis and management had increased, and they felt less lonely.

**Table:** 

Table 1	BECI average score	WEMWBS average score
Before the group	36.6	41.3 indicative of possible/mild depression
After the group	30.4	47.1 indicative of average wellbeing

Table 1

**Conclusions:**

A psychoeducational group for carers was well received by participants, and on average they experienced an improvement on the burden associated to caregiving and their wellbeing. Analysis of results was limited due to drop outs, but their feedback included that they did not feel the need anymore for this intervention. Further feedback from participants has contributed to change the sessions’ content based on co-production, and the time of the group to enable attendance.

**Disclosure of Interest:**

None Declared

